# Intravenous versus oral iron for iron deficiency anaemia in pregnant Nigerian women (IVON): study protocol for a randomised hybrid effectiveness-implementation trial

**DOI:** 10.1186/s13063-022-06690-2

**Published:** 2022-09-08

**Authors:** Bosede B. Afolabi, Ochuwa A. Babah, Opeyemi R. Akinajo, Victoria O. Adaramoye, Titilope A. Adeyemo, Mobolanle Balogun, Aduragbemi Banke-Thomas, Rachel A. Quao, Gbenga Olorunfemi, Ajibola I. Abioye, Hadiza S. Galadanci, Nadia A. Sam-Agudu

**Affiliations:** 1 Department of Obstetrics & Gynaecology, Faculty of Clinical Sciences, College of Medicine, University of Lagos, P.M.B. 12003, Idi-Araba, Lagos, Nigeria; 2 Department of Obstetrics and Gynaecology, Lagos University Teaching Hospital, Idi-Araba, Lagos, Nigeria; 3 Department of Haematology & Blood Transfusion, Faculty of Clinical Sciences, College of Medicine, University of Lagos and Lagos University Teaching Hospital, Idi-Araba, Lagos, Nigeria; 4 Department of Community Health & Primary Care, College of Medicine, University of Lagos, Lagos, Nigeria; 5 Global Maternal and Newborn Health Hub, Institute of Lifecourse Development, University of Greenwich, London, UK; 6 IVON trial, College of Medicine, University of Lagos, Idi-Araba, Lagos, Nigeria; 7 Division of Epidemiology and Biostatistics, School of Public Health, University of Witwatersand, Johannesburg, South Africa; 8 Department of Global Health and Population, Harvard T.H. Chan School of Public Health, Boston, MA USA; 9 African Center of Excellence for Population health and Policy, Bayero University Kano, Kano, Nigeria; 10 Department of Obstetrics and Gynaecology, College of Health Sciences Bayero University Kano/Aminu Kano Teaching Hospital, Kano, Nigeria; 11 International Research Center of Excellence, Institute of Human Virology Nigeria, Abuja, Nigeria; 12 Institute of Human Virology, University of Maryland School of Medicine, Baltimore, USA

**Keywords:** Anaemia, iron deficiency, Anaemia in pregnancy, Pregnancy, Ferric carboxymaltose, Ferrous sulphate, Depression, Cost-effectiveness, Implementation science, Protocol

## Abstract

**Background:**

Anaemia in pregnancy is highly prevalent in African countries. High-dose oral iron is the current recommended treatment for pregnancy-related iron deficiency anaemia (IDA) in Nigeria and other African countries. This oral regimen is often poorly tolerated and has several side effects. Parenteral iron preparations are now available for the treatment of IDA in pregnancy but not widely used in Africa.

The IVON trial is investigating the comparative effectiveness and safety of intravenous ferric carboxymaltose versus oral ferrous sulphate standard-of-care for pregnancy-related IDA in Nigeria. We will also measure the implementation outcomes of acceptability, feasibility, fidelity, and cost-effectiveness for intravenous ferric carboxymaltose.

**Methods:**

This is an open-label randomised controlled trial with a hybrid type 1 effectiveness-implementation design, conducted at 10 health facilities in Kano (Northern) and Lagos (Southern) states in Nigeria. A total of 1056 pregnant women at 20–32 weeks’ gestational age with moderate or severe anaemia (Hb < 10g/dl) will be randomised 1:1 into two groups. The interventional treatment is one 1000-mg dose of intravenous ferric carboxymaltose at enrolment; the control treatment is thrice daily oral ferrous sulphate (195 mg elemental iron daily), from enrolment till 6 weeks postpartum.

Primary outcome measures are (1) the prevalence of maternal anaemia at 36 weeks and (2) infant preterm birth (<37 weeks’ gestation) and will be analysed by intention-to-treat. Maternal full blood count and iron panel will be assayed at 4 weeks post-enrolment, 36 weeks’ gestation, delivery, and 6 weeks postpartum. Implementation outcomes of acceptability, feasibility, fidelity, and cost will be assessed with structured questionnaires, key informant interviews, and focus group discussions.

**Discussion:**

The IVON trial could provide both effectiveness and implementation evidence to guide policy for integration and uptake of intravenous iron for treating anaemia in pregnancy in Nigeria and similar resource-limited, high-burden settings. If found effective, further studies exploring different intravenous iron doses are planned.

**Trial registration:**

ISRCTN registry ISRCTN63484804. Registered on 10 December 2020

Clinicaltrials.govNCT04976179. Registered on 26 July 2021

The current protocol version is version 2.1 (080/080/2021).

**Supplementary Information:**

The online version contains supplementary material available at 10.1186/s13063-022-06690-2.

## Background

Anaemia in pregnancy (AIP), defined as haemoglobin concentration of <11 g/dl among pregnant women [1], remains a significant global health challenge, especially in resource-limited settings [2]. AIP is caused by several conditions including nutritional disorders, malaria, infection, haemoglobinopathies, haemorrhage, and chronic disease. However, iron deficiency anaemia (IDA) is the commonest cause of AIP, accounting for 50–75% of diagnoses [3]. AIP has significant implications on both maternal and neonatal outcomes, leading to an increased risk of maternal morbidity and mortality, neonatal low birth weight, and prematurity [4, 5]. AIP has also been associated with an increased risk of maternal depression [6, 7]. These complications can be prevented if maternal anaemia is promptly and adequately treated.

AIP is estimated to affect nearly 50% of pregnant women in Africa [3, 8]. Nigeria is the most populous country in Africa and bears a significant burden of anaemia among pregnant women with an estimated prevalence of 20–40% [8, 9]. Earlier studies in South-East and South-South Nigeria found the prevalence of AIP between 20 and 76% [10–13]. A systematic review reported the prevalence of IDA between 25 and 45.6% in Nigeria [14], while a recent study in Lagos, South-West Nigeria, reported an IDA prevalence of 12.3%, using stringent values of Hb < 10g/dl and ferritin < 15μg/l [15].

While serum ferritin concentration is considered a cost-effective first-line diagnostic tool for IDA (Peyrin), it is not always readily available in LMIC because of the general poor access to laboratory services [16]. Therefore, as IDA is the commonest cause of AIP, it is acceptable to treat AIP with iron therapy especially when anaemia is moderate or severe, without a laboratory confirmation of iron deficiency. However, as there are still other significant causes of AIP including infections and other nutritional deficiencies, it is important to determine the true incidence of IDA in pregnancy in our environment and the safety and efficacy of the intervention in IDA specifically, as well as in moderate to severe AIP in general.

Currently, global recommendations for the management of AIP advise that pregnant women be treated with high-dose oral iron at 120 mg of elemental iron daily until haemoglobin rises to normal (Hb 11.0g/dl or higher) [17]. However, oral iron has several disadvantages. First, high doses are often poorly tolerated due to significant gastrointestinal adverse effects such as vomiting, constipation, diarrhoea, and abdominal pain, limiting treatment adherence [3, 18]. In addition, the thrice daily dosing that is recommended to treat mild to moderate AIP in Nigeria [19] is inconvenient and difficult to complete as prescribed. Finally, IDA management with oral iron during pregnancy requires close, continuous monitoring, and in settings with inadequate health-seeking behaviour and economic limitations, few women complete the recommended course of oral iron, leading to an increased risk of undertreated and persistent IDA in pregnancy. Rates of completion for prescribed oral iron regimens for AIP in Nigeria and other African countries have been reported between 34 and 58% [20]. Nigeria thus presents an appropriate setting in which to assess the effectiveness and safety of an alternate treatment for reducing the burden and negative impact of AIP.

Intravenous iron is an alternative to oral iron supplementation. It requires minimal client-facility contact and corrects anaemia faster than oral preparations [21, 22]. Newer intravenous iron preparations have been found to be well-tolerated and with fewer adverse effects than high molecular weight iron dextran [23]. In Nigeria, pregnant women seek antenatal care relatively late, with most first-time visits in the fourth or fifth month of pregnancy, and less than 60% attend four or more antenatal visits as recommended by the World Health Organization [24]. Thus, the use of a conveniently dosed iron formulation that is safe, rapid-acting, and cost-effective can improve completion rates and outcomes for IDA. Ferric carboxymaltose (FCM) is known to be safe and effective for use in pregnancy, and a full treatment dose can be given in 1–2 visits [25–27].

The IVON trial aims to examine the effectiveness and safety of parenteral versus oral iron in the Nigerian context. The trial adopts an implementation science approach to understand the implementation climate for this relatively new treatment and to determine its cost-effectiveness in the Nigerian health system. Specifically, the objectives of the IVON trial are:

1. To determine the effect of intravenous FCM on the prevalence of maternal anaemia at 36 weeks’ gestation, and on the incidence of preterm birth, compared with oral ferrous sulphate (FS)
2. To determine the effect of intravenous FCM on increase in maternal haemoglobin concentration 4 weeks after administration, and on maternal clinical outcomes including postpartum haemorrhage, sepsis, shock, need for blood transfusion, and prevalence of depression, compared with oral FS
3. To determine the effect of intravenous FCM on the incidence of low infant birthweight, stillbirth, and neonatal mortality and on infant breastfeeding and immunisation at 6 weeks of life, compared with oral FS
4. To measure implementation outcomes relating implementation of intravenous FCM, including acceptability, feasibility, and fidelity of administration in the Nigerian context
5. To determine the cost-effectiveness of intravenous FCM compared with oral FS in the treatment of IDA in pregnancy in Nigeria

## Consultations and formative research

Before study initiation, the IVON team engaged and consulted with government officials overseeing maternal and child health programmes in the study states of Lagos and Kano in South-West and North-West Nigeria, respectively. The officials provided guidance on the study approach to suit the health system context and how study findings may be translated into health policy and routine practice.

Before finalising the study protocol, we conducted a formative assessment. We used the Consolidated Framework for Implementation Research (CFIR) [28] to inform the design of the formative study, which assessed for barriers and facilitators of successful intervention implementation in our Nigerian study settings. We explored determinants along the five CFIR domains: characteristics of the intervention, individuals involved, outer setting, inner setting, and implementation process. We conducted 12 focus group discussions and 53 in-depth interviews among 193 participants in Kano and Lagos states, including pregnant women, male partners, healthcare workers, and policymakers [29]. Key findings and relevant design adjustments are summarised in Table 1.

**Table 1:** Team-ratified changes for the IVON study design based on formative findings

Original study design	Formative assessment findings	Adjusted study design	CFIR category of change
No standard script for patient counselling by healthcare workers	Threat of misinformation and conspiracy theories	Standard patient information script to be used for all IV iron administrations	• Individuals involved
Participants only receive a signed consent form	Important to provide clear, standard reference information for both participants and family members	Participants and family to receive an illustrated flyer in simple English or local language describing the IV iron procedure, uses, and benefits	• Individuals involved • Outer setting
Expert trainers provide training only at baseline; step-down training thereafter	HCWs need expert periodic training given the novelty of the intervention	Expert trainers to provide both baseline and periodic refresher training to HCWs every 6 months	• Inner setting • Individuals involved
Study implementation focused on intervention, patient, healthcare workers, clinic	The study needs to consider all social levels for intervention success	Both study implementation and dissemination to be conducted with relevant socioecological model applications	• Implementation process

## Methods

### Study design

**Fig. 1:**
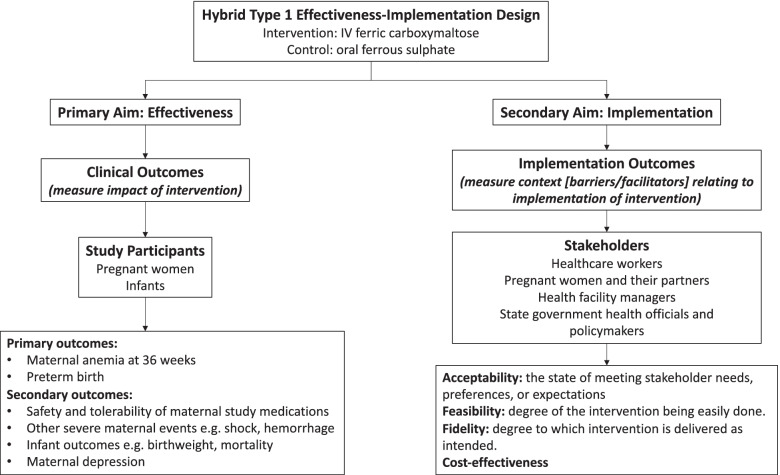
IVON trial effectiveness-implementation design

The IVON trial will adopt a hybrid type 1 effectiveness-implementation design, where the effect of an intervention is evaluated while documenting observations about its implementation [30]. The effectiveness study is a two-arm, open-label, individually randomised controlled trial (RCT) that assesses the comparative effectiveness and safety of FCM versus oral iron in treating IDA among pregnant Nigerian women. Intervention effectiveness will be assessed through analyses for the primary and secondary measures, while implementation will be documented through reporting on the implementation outcomes of (1) acceptability, (2) feasibility, (3) fidelity, and (4) cost-effectiveness (Fig. 1).

We used the SPIRIT checklist for protocol reporting [31] to guide the development of our protocol (Additional file 1).

### Study setting and sites

**Fig. 2:**
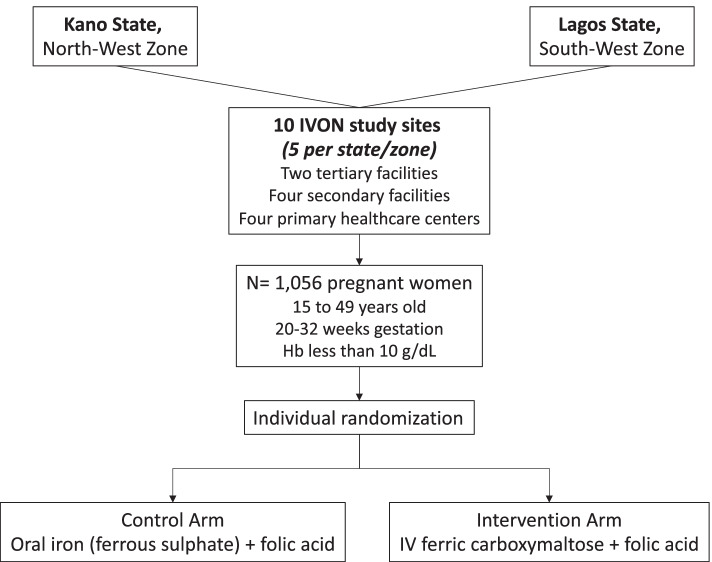
IVON clinical trial flowchart

The study will be conducted at health facilities in Kano and Lagos states, representing the North-West and South-West zones, respectively, which, by population, are the two largest states in Nigeria. Rates of antenatal care utilisation are 63.4% and 51.4% in urban and rural areas of Kano, respectively [32], and 81.4% in Lagos [33]; estimates for delivery by skilled personnel are 21.5% and 83.6% in Kano and Lagos, respectively [34]. The prevalence of AIP in Kano and Lagos is reported at 17.5 to 75% [35, 36] and 35.3 to 87.2% [37, 38], respectively. Five study sites (one tertiary, two secondary, and two primary health facilities) were selected per state, for a total of 10 sites (Fig. 2). Site eligibility criteria were exclusively publicly funded primary, secondary, or tertiary health facility; antenatal clinic attendance of at least 60 pregnant women per month; delivery rate of at least 20 women per month; consistent 24-h routine vaginal delivery services; and onsite testing for Hb, HIV, and malaria.

### Study population and eligibility criteria

#### Inclusion criteria

1. Pregnant women aged 15 to 49 years old between 20 and 32 weeks’ gestation
2. Moderate or severe anaemia (Hb <10 g/dl), since evidence supports that most anaemic pregnant women have IDA [3]. The alternative action of awaiting laboratory confirmation of IDA takes a minimum of 48 h, which may affect enrolment rates for the trial

#### Exclusion criteria

Pregnant women with:

1. Medically confirmed significant bleeding, major surgery, or blood transfusion within the last 3 months
2. Severe symptomatic anaemia requiring blood transfusion
3. Anaemia confirmed to be from other known causes besides IDA, e.g. sickle cell anaemia
4. Clinically confirmed malabsorption syndrome
5. Hypersensitivity or known allergy to any forms of iron or known severe drug allergies
6. Severe allergic reactions such as severe asthma
7. History of autoimmune disease, e.g. systemic lupus erythematosus, rheumatoid arthritis
8. Pre-existing maternal depression or other psychiatric illness

### Intervention and control trial drugs

The intervention drug under investigation is intravenous ferric carboxymaltose (Ferinject®, FCM), and the standard-of-care drug is oral ferrous sulphate (Fesulf®). FCM is given as a single dose of 20 mg/kg up to a maximum of 1000 mg diluted in 200 ml of 0.9% sodium chloride and infused over 15–20 min. FS will be given as one 200-mg tablet containing 65 mg of elemental iron, three times daily, to be taken 1 h before meals or 2 h after meals until 6 weeks postpartum. Women in both intervention and control groups will also receive 5 mg of folic acid and 100 mg of vitamin C daily throughout pregnancy. In addition, participants in both groups will receive daily reminders via short message service (SMS) sent directly to their mobile phone numbers, to take their oral medications. Pill counts will also be done at each study visit.

### Trial outcomes

#### Effectiveness outcomes

Primary outcomes are the prevalence of maternal anaemia (Hb <10 g/dl) at 36 weeks’ gestation and the incidence of preterm birth (<37 weeks’ gestation) (Fig. 1). Secondary maternal outcomes include an increase in maternal haemoglobin concentration 4 weeks after administration; the prevalence of maternal depression at 36 weeks’ gestation and at 2 weeks postpartum as assessed with the Edinburgh Postnatal Depression Scale (EPDS); safety of trial drugs as determined by the prevalence of severe adverse events; incidence of severe maternal events such as shock and sepsis as determined by physician diagnosis or medical record documentation; incidence of postpartum haemorrhage determined by visually estimated blood loss >500 ml for vaginal delivery and >1000 ml for caesarean section; and need for blood transfusion. Secondary infant outcomes include incidence of low birthweight (<2.5 kg); prevalence of stillbirth; small-for-gestational age (birthweight below the 10th percentile for gestational age), neonatal death (within 28 days of birth); and proportion of infants being breastfed at 1, 2, and 4 weeks of life, and having received Bacille–Calmette–Guerin, oral polio, and hepatitis vaccinations within 4 weeks of life (Table 2).

**Table 2:** Specifically defined study outcomes

Effectiveness outcomes				
Domain	Specific measurement	Specific metric	Method of aggregation	Time point
**Primary outcomes**				
Maternal anaemia	Haemoglobin concentration in g/dl measured with a haematological auto-analyser	Value at 36 weeks’ gestation	Proportion of participants with Hb <11 g/dl	36 weeks’ gestation
Preterm birth	Gestational age in weeks at delivery	Value at delivery	Proportion of preterm deliveries below 37 weeks 0 days gestation	Date of delivery
**Secondary maternal outcomes**				
Maternal haemoglobin concentration	Haemoglobin concentration measured in g/dl with a haematological auto-analyser	Change in value at 4 weeks after enrolment	Mean change in haemoglobin concentration	Four weeks after enrolment
Maternal depression	Edinburgh Postnatal Depression Scale (EPDS)	Value at 36 weeks’ gestation and at 2 weeks postpartum	Proportion of participants with score ≥10 on the EPDS	36 weeks’ gestation and 2 weeks postpartum
Severe adverse events (grade 3 or more)	Physician diagnosis or medical record documentation or report from participants	Value at any time point after enrolment	Proportion of participants with serious adverse events	Any time point after enrolment
Severe maternal events such as shock and sepsis	Physician diagnosis or medical record documentation	Value at any time point after enrolment	Proportion of participants with severe maternal events	Any time point after enrolment
Postpartum haemorrhage	Estimated blood loss in ml measured visually or by physician diagnosis of need for blood transfusion	Value at delivery	Proportion of participants with estimated blood loss >500 ml for vaginal delivery and >1000 ml for caesarean section/proportion of participants in need of blood transfusion	At delivery and within 24 h after
**Secondary infant outcomes**				
Low birth weight	Baby’s weight in grammes at delivery	Value at delivery	Incidence of low birth weight (<2.5 kg)	At delivery
Stillbirth	Physician diagnosis, medical records, or report from a participant	Value at birth	Proportion of infants who die after 28 weeks’ gestation but before or during delivery	At delivery
Small-for-gestational age	Baby’s weight in grammes at delivery	Value at birth	Proportion of infants with birth weight below the 10th percentile for gestational age	At delivery
Neonatal death	Physician diagnosis or medical record documentation	Value within 28 days of delivery	Proportion of infants who die within 28 days of birth	From delivery to 28 days of life
Breastfed infants	Medical record documentation or report from participants	Value within 4 weeks of birth	Proportion of infants being breastfed at 1, 2, and 4 weeks of life	At 4 weeks after delivery
Early neonatal vaccinations	Medical record documentation	Value within 4 weeks of birth	Proportion of infants who have received Bacille–Calmette–Guerin, oral polio, and hepatitis within 4 weeks of birth	At 4 weeks after delivery
**Implementation outcomes and measures of cost-effectiveness**				
Acceptability of intervention	Acceptability of Intervention measure (AIM) survey tool, FDGs, KIIs	Value at baseline and end of the study	Degree of acceptability of intervention by target stakeholders	End of study
Feasibility of intervention	Feasibility of Intervention Measure (FIM) survey tool, FDGs	Value at baseline and end of the study	Degree of feasibility of carrying out intervention by health care workers	End of study
Fidelity of intervention	Intervention procedure checklist	Value during study	Degree of fidelity of carrying out intervention by health care workers	End of study
Costs of intervention and control treatment	Survey, hospital records on cost of services	Value during study	Cost-effectiveness analysis for the study intervention from limited societal perspective	End of study

A score of ≥10 on the Edinburg Postnatal Depression scale (EPDS) will be considered indicative of postpartum depression, as this has been shown to be highly sensitive for diagnosing minor depression (Table 2) [39]. All women screened positive for depression will be referred to the collaborating psychiatrist for further assessment.

#### Implementation outcomes and measures of cost-effectiveness

Implementation outcomes to be assessed are acceptability (the state of meeting stakeholder needs, preferences, or expectations), feasibility (the state or degree of the intervention being easily or conveniently done), and fidelity (the degree to which the intervention is delivered as intended). In addition, costs of the intervention and control treatment options to pregnant women and to the health system will be collected (Table 2).

### Participant recruitment

**Fig. 3:**
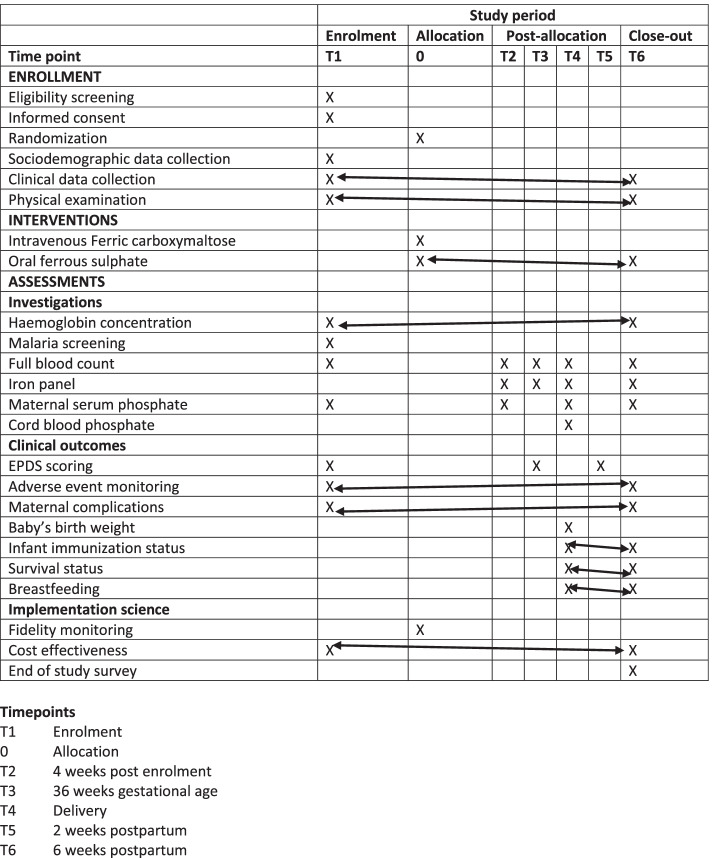
Spirit figure showing specific time points in IVON trials. Note:  denotes variables that will be assessed at each visit from the selected time point (X) to the end point depicted by the double-headed arrow. Acceptability of intervention will be assessed pre-trial, and findings used in protocol modification

Participants will be enrolled at 20–32 weeks’ gestational age and followed up until 6 weeks postpartum (Fig. 3). Pregnant women presenting to antenatal care clinics at study sites will be approached by research nurses and counselled on anaemia in pregnancy and the IVON trial. Consenting pregnant women will be screened for Hb by finger pinprick, using the Hemocue® haemoglobinometer. Women with Hb < 10g/dl who meet other eligibility criteria will be further counselled about the study, including risks, benefits, and what is expected as a participant, as part of the informed consent process. Potential participants will be able to sign informed consent forms immediately or to sign later if they wish to discuss with male partners or family members.

Site research nurses and coordinators will all be required to maintain Good Clinical Practice certification and will be trained on the research protocol. Recruitment is targeted for completion by end of July 2023.

### Sample size estimation

Per the multi-country FER-ASAP (FERric carboxymaltose-Assessment of SAfety and efficacy in Pregnancy) study in Europe, Asia, and Australia [40], the expected levels of anaemia correction for oral iron and FCM in pregnancy are approximately 70% and 84%, respectively. At 5% significance, 90% power and adjusting for 15% attrition [41], 1056 pregnant women (528 in each study arm) was the estimated sample size to detect a 14% difference in the correction of AIP between the control (70%) and intervention groups (84%).

### Randomisation and allocation

Eligible participants will undergo individual randomisation and allocation. Randomisation and concealment will be done by Sealed Envelope, UK (www.sealedenvelope.com), through a web-based randomisation service, that will randomise in a 1:1 ratio in blocks stratified according to the study site. Once consent is signed, the research nurse enters enrolment details such as biodata and obstetric history of the participant into an electronic handheld tablet. On submission of the electronic form, the Sealed Envelope software will randomly allocate the participant to intervention or control.

### Blinding

There is no blinding except during the data analysis. Given the obvious morphological differences of the intervention (intravenous solution) and control (oral tablet) drugs, both participants and study staff will be aware of study arm allocations following randomisation. Statisticians will be blinded to treatment assignment during analysis.

### Data collection

For data on effectiveness outcomes, following randomisation, data are collected electronically, using case report forms designed with REDcap electronic data capture tools, hosted at the College of Medicine, University of Lagos [42, 43]. General and obstetric physical examinations are performed at baseline and at each study visit from enrolment to 6 weeks postpartum. Hb and full blood count measurements are done for all study visits and for five of six visits, respectively. Malaria is assessed with a rapid diagnostic test (SD BIOLINE Malaria Ag P.f) at enrolment and whenever participants have symptoms consistent with malaria. All participants diagnosed with malaria are treated with Arthemeter-Lumefantrine as per national guidelines [44]. Serum ferritin alone is done at enrolment, but complete iron profile comprising serum ferritin, serum iron, total iron binding capacity, and transferrin saturation are done at 4 weeks after initiation of the study drug, at 36 weeks, at delivery, and at 6 weeks postpartum. Maternal serum phosphate level is assayed at enrolment, 4 weeks after iron infusion/oral iron commencement, on the day of delivery, and at 6 weeks postpartum. Cord blood phosphate is assayed on the day of delivery. Participants are seen in a routine antenatal clinic every 4 weeks till 28 weeks’ gestation, and every 2 weeks until 36 weeks, then weekly until delivery. Thereafter, they are seen at 2 and 6 weeks postpartum (Fig. 3).

To minimise loss-to-follow up in the RCT, retention counselling for study participants will be extended to male partners and relevant family members during antenatal visits and will emphasize facility delivery at study sites. Also, verification of locator information (phone numbers and residential addresses) will be done at every clinic visit. Finally, research nurses will send SMS appointment reminders to participants, who will also be tracked in the community (at their homes or other places of delivery, if applicable), when they do not show up for expected study visits.

For data on implementation outcomes, besides the CFIR-guided qualitative data collected during the formative study, quantitative data on acceptability and feasibility was collected from among healthcare workers, using the Acceptability of Intervention Measure (AIM) and Feasibility of Intervention Measure (FIM) tools developed by Weiner et al. [45]. For the trial proper, concurrent qualitative and quantitative assessments of acceptability and feasibility will be repeated at the end of the study. Exit interviews will be conducted quantitatively through surveys among 25% randomly selected participants in each arm of the trial and qualitatively through in-depth interviews using semi-structured interview guides among purposively selected participants (Table 3).

**Table 3:** Timeline of data collection for implementation outcomes

Implementation outcome	Data collection time point(s)			Target stakeholders	Mode of data collection
	Baseline (formative assessments)	During	End of study		
Acceptability (includes facilitators and barriers)	X		X	Healthcare workers, pregnant women and their partners, health facility managers, state government health officials, policymakers	AIM survey and site assessment tool, FGDs, KIIs
Feasibility	X	X	X	Healthcare workers	FIM survey tool, FGDs
Fidelity		X		Healthcare workers (only those delivering intervention)	Intervention procedure checklist and in-depth interview
Cost-effectiveness		X	X	Pregnant women, healthcare workers, hospital administrators	Cost survey and logbook

*AIM* acceptability of intervention measure, *FIM* feasibility of intervention measure, *FGD* focus group discussions, *KII* key informant interview

For cost data needed for the cost-effectiveness analysis, direct costs such as cost of medications including the intravenous FCM and oral FS, folic acid, and vitamin C, out-of-pocket expenses by users including transportation to care, opportunity costs of antenatal visits, short admission stay for infusion of intravenous FCM and for management of any complications and health system costs such as laboratory consumables, supplies, and diagnostics will be collected. Patient cost data will be collected from IVON trial participants at follow-up visits during the antenatal and postpartum periods. Health system costs will be derived from hospital administrators and from data available in published studies from Nigeria.

### Data management

Data collection and reporting for both effectiveness and implementation outcomes will be guided by standard operating procedures that will be periodically updated, shared with, and communicated to the study staff. Effectiveness data will be captured in electronic case report forms designed for each study visit, using assigned codes, and uploaded in real time to the central REDcap server after verification by each site coordinator. Implementation data will be captured in standard study tools or as transcripts in the study database, which is password protected. For confidentiality and privacy, all qualitative and quantitative participant data will be de-identified from the point of data collection to curation and publication, and only the principal investigator, project manager, and data manager will have access to the study data.

### Statistical methods and analyses

#### Interim analysis and conditions for early trial termination

An interim analysis will be conducted by the Data and Safety Monitoring Committee after 50% of study participants have been recruited and fully followed up, with the objective to (1) evaluate the overall safety of the interventional intravenous iron; (2) examine the adequacy of assumptions made for sample size estimation; (3) decide, based on clear evidence of efficacy or lack thereof, whether early termination of the trial is warranted; and (4) determine whether there are unanticipated reasons for early trial termination or modification. There will be considerations for study discontinuation if there is a ≥28% difference (double the 14% assumed difference) in the prevalence of women with improved anaemia in the intervention group compared to the control group.

#### Quantitative analysis for effectiveness measures

Categorical variables will be expressed as frequencies and percentages. For continuous variables, a Shapiro–Wilk test of normality will be performed, and normally distributed data will be presented as means with standard deviations, while non-normally distributed data will be presented as medians with interquartile ranges. The main analysis will be by intention-to-treat. The intention-to-treat (ITT) population will include all subjects who were enrolled and randomised. Baseline characteristics will be compared between the intervention and control groups using Pearson’s chi-square (for categorical variables), Student’s *t*-test (for normally distributed continuous variables), or Mann–Whitney *U* (for non-normally distributed continuous variables). Paired *t*-test or Wilcoxon signed rank *t*-test will be used to assess the intra-group change in Hb, ferritin, and transferrin saturation concentrations from baseline to 6 weeks postpartum.

Univariable and multivariable binary logistic regression will be used to compare the proportions of women achieving anaemia correction, with the assigned study group as the primary explanatory variable. Simple and multiple linear regression modelling will assess changes in continuous variables. Kaplan–Meir plots and unstratified log-rank test will be used to explore time-to-anaemia correction.

The above analysis of the proportions achieving anaemia correction and time-to-anaemia correction will be repeated in subgroups of baseline iron deficiency (versus no iron deficiency) and iron deficiency anaemia (versus no iron deficiency anaemia). Effect modification of the effectiveness of interventions by baseline iron deficiency and iron deficiency anaemia will be evaluated using the likelihood ratio tests and *p*-values reported. Iron deficiency will be defined in accordance with the World Health Organization guideline as serum ferritin < 15μg/l (ref), and iron deficiency anaemia will be defined as iron deficiency in the presence of anaemia.

Sensitivity analysis will be conducted in the modified intention-to-treat population. The modified intention-to-treat population will include participants who complete 36 weeks of delivery and are eligible for assessment of the primary endpoint. Two-tailed test of hypothesis and 95% confidence interval is the assumed statistical level of significance. Statistical analysis will be conducted using STATA SE version 16.0 (STATA Corp, TX, USA) software.

#### Analyses of implementation outcomes

All qualitative interviews will be recorded and transcribed verbatim. Transcribed data will be coded both deductively (informed by CFIR) [46] and inductively, to generate further themes from the data. Content analysis will then be used to capture underlying ideas and patterns.

AIM and FIM survey responses will be interpreted per Weiner et al. guidance [45]. Fidelity will be calculated as a proportion of IV iron administrations performed that met the minimum set of checklist items for each FCM administration completed. The analysis will be done using descriptive statistics and test of difference regressions.

#### Cost-effectiveness analysis

Measures of cost and effectiveness will be obtained using primary client-level data on cost, hospital costs for providing care, and results of clinical outcomes from the effectiveness evaluation. Specifically, cost elements of both oral and intravenous iron treatment options will be quantified and comparatively assessed at an individual client level, using a decision tree [47, 48]. Comparative analyses of direct medical costs of intravenous FCM and oral ferrous sulphate costs and costs for administration will be included. Analysis of all costs borne by clients from potential adverse events, and from any loss of productivity while incapacitated by these adverse events, will be conducted for all affected participants. Empirical evidence on probabilities will be obtained from the trial as well as from secondary data sources.

Analysis will be conducted from a limited societal perspective [49]. The incremental cost-effectiveness ratio (ICER) will be obtained from mean values for costs and effectiveness, the latter defined by significant differences in the prevalence of anaemia at 36 weeks, and in the incidence of preterm birth. The confidence interval for the ICER will be derived using non-parametric bootstrapping to quantify sampling uncertainty. Uncertainty will be addressed by a univariate sensitivity analysis, which aims to identify parameters whose uncertainty has the largest influence on the ICER. In addition, a probabilistic sensitivity analysis, in which all parameters with uncertainty will be varied within their confidence intervals, will provide a credible time interval for the estimated result of the ICER. No discounting will be incorporated in the analysis as the analytic horizon is less than a year.

### Data monitoring

IVON has an Administrative Core, a Steering Committee, Clinical Trial Monitors, and a Data and Safety Monitoring Committee (DSMC). The Administrative Core comprises the principal investigator and a full-time project manager who provide administrative oversight, manage research activities, and coordinate all internal (implementing staff) and external (collaborator) meetings. The Steering Committee includes the principal investigator, all co-investigators, and site coordinators; provides cross-functional leadership and direction and project governance; and ensures adherence to the research protocol to facilitate the achievement of project goals. Clinical Trial Monitors will conduct weekly site monitoring visits to ensure and verify human subject protection and conduct documentation audits and verification of reported trial data.

The DSMC is an independent, five-person external monitoring committee including an obstetrician, a haematologist, a statistician, an experienced trial pharmacist, and a paediatrician with research expertise. The DSMC met once before the trial started and will meet every 6 months during the study. The DSMC will meet after the 50th participant has completed the study to assess the progress of the RCT, recruitment rate, and effect of drugs and decide whether to modify, terminate, or continue the clinical trial.

### Adverse event monitoring

In our study, an adverse event (AE) will be defined as any untoward medical occurrence in a participant regardless of the possibility of a causal relationship to the trial drug. All adverse events that occur from the point of enrolment and throughout the study duration will be collected non-systematically from each participant. Participants will be routinely asked at every study visit “do you have any symptoms now or noticed any since your last visit?” If a participant reports an AE at enrolment but before the administration of study intervention, the AE will be reported as not related to the trial drug. All adverse events reported after enrolment into the study and until the end of the study will be defined and graded for severity using the US National Cancer Institute Common Terminology Criteria for Adverse Events version 5.0 [50] and documented as such for analysis. The suspected relationship to trial drugs will be determined by the principal investigator and documented along with the interventions given, and the outcome.

A serious adverse event (SAE) is any event which is fatal or life-threatening, or results in persistent or significant disability/incapacity, or constitutes a congenital anomaly/birth defect, or requires inpatient hospitalization or prolongation of existing hospitalization. Other criteria include an event that is sufficiently significant as to require medical or surgical intervention to prevent one of these outcomes or is a significant hazard as determined by the Data Safety Monitoring Board. An adverse event that meets the criteria for a SAE between enrolment and end of the study will be reported to the local Institutional Review Board (IRB) as an SAE. Discontinuation of the study drug may be considered if a participant experiences a severe adverse drug event of grade 3 or higher [50].

Decision for discontinuation will be made by the principal investigator and/or the study participant. Study personnel will document the circumstances and data leading to the discontinuation. Investigators will also determine whether an AE is a suspected unexpected serious adverse reaction (SUSAR) given the participants’ clinical course, previous medical conditions, and concomitant medications. A SUSAR is defined as an untoward and unintended response to a trial drug, which is not listed in the product information, and meets one of the following serious criteria: results in death, is life-threatening, requires hospitalization or prolongation of an existing hospitalization, results in persistent or significant disability or incapacity, or is a congenital anomaly or birth defect. SUSAR report will be expeditiously submitted to health regulatory authorities. The costs of managing all adverse drug events related to trial drugs will be borne by the study.

Using an intention-to-treat analysis, the absolute risk, the frequency/incident rate, and severity grade of anticipated adverse drug reactions such as abdominal pain, nausea and vomiting, hypotension, and hypophosphatemia for participants in each study arm, the incident rate of any serious adverse reactions (SAR) and suspected unexpected serious adverse reaction (SUSAR) for each arm of the study will be determined. Serious adverse reactions (SARs) are SAEs that are thought to be causally linked to the trial drug. The mean and SDs or median and interquartile range for serum phosphate level will be calculated as appropriate, while extreme values will be stated if observed.

## Discussion

At present, there is a paucity of effectiveness-implementation evidence on the use of intravenous iron for IDA in pregnancy in Nigeria and other African countries [51, 52]. In this regard, the IVON trial is a pioneer hybrid design trial whose findings are expected to have significant ramifications for maternal and neonatal health pertaining to AIP in Nigeria, West Africa, and the rest of Africa.

### Strengths

The IVON trial takes an implementation science-informed approach to understanding issues relating to the use of intravenous iron in treating IDA in pregnancy at different social-ecological levels in Nigeria. This includes evaluation of implementation outcomes at the individual (pregnant women), interpersonal (male partners, family), health facility, community-health system relationship, and state/federal government levels. Additionally, our assessment of cost-effectiveness, and more broadly, value-for-money of the intervention, will provide critical information for strategic decision-making by governments and partners [53]. The trial has the potential to support realisation of Sustainable Development Goal (SDG) 2, which aims to improve nutritional deficiencies, of which iron deficiency is a major component, and SDG 3, which targets good health for all [54], by its focus on AIP and maternal and perinatal morbidity and mortality as well as on maternal mental health, an often-neglected outcome.

### Potential limitations and future studies

The IVON trial does not involve all states or geo-political zones in Nigeria, and as such, its findings may not be generalizable to the entire country. However, it covers its two largest cities and involves participants and facilities in both rural and urban areas in the North and South of the country. Furthermore, while the trial assesses for the effectiveness of IV iron, it does not assess for the effectiveness of different dosing strengths or schedules. In some studies, IV iron has been given in more than one dose, for example, a multisite international study and another in India [55, 56], while for studies in Bangladesh and Australia, one dose was given [57, 58]. Also, a single dose of up to 1500 mg has been given safely in another Indian study [59]. In settings like Nigeria, where antenatal care clinic attendance is poor, a highly effective and safe single dose will be desirable, thus necessitating studies to examine this.

Findings from the IVON trial will be disseminated among stakeholders within and outside Nigeria, taking levels of clinical and scientific expertise into consideration. Local dissemination channels will include the platform of the Nigeria Implementation Science Alliance [60], which gathers academia, public health programme implementers, community members, and government officials to review and discuss evidence-to-practice for health conditions of major importance in the country.

### Trial status

Participant recruitment started on 9 August 2021 and is projected to end by 10 July 2023.

## Supplementary Information

**Additional file 1.** SPIRIT checklist.

**Additional file 2.** Ethics approval and consent to participate.

## Data Availability

We will store the data in the Open Science Framework after obtaining approval from the ethics committees. No client identifier will be included in the data to be shared.

## Abbreviations

AIP: Anaemia in pregnancy
DSMC: Data and Safety Management Committee
EPDS: Edinburg Postnatal Depression scale
FCM: Ferric carboxymaltose
FGD: Focus group discussion
FIM: Feasibility intervention measure
FS: Ferrous sulphate
Hb: Haemoglobin
HCP: Health care provider
ICER: Incremental cost-effectiveness ratio
IDA: Iron deficiency anaemia
KII: Key informant interview
RCT: Randomised control trial
SDGs: Sustainable development goals
SMS: Short message service
USA: United States of America
