# Supplementary material for: Intravenous versus oral iron for iron deficiency anaemia in pregnant Nigerian women (IVON): study protocol for a randomised hybrid effectiveness-implementation trial
Source: Trials. 2022 Sep 8;23:763. doi: 10.1186/s13063-022-06690-2 (PMC9454388; doi:10.1186/s13063-022-06690-2)
Supplement: Supplementary file 2 — Additional file 2. Ethics approval and consent to participate. [file 13063_2022_6690_MOESM2_ESM.doc]

**IVON TRIAL**

**PROCESSES OF RECRUITING POTENTIAL PARTICIPANTS AND OBTAINING INFORMED CONSENT**

On each clinic day at the study sites, the research nurse gives group counselling to all women presenting to the antenatal clinic to create awareness about the IVON trial. Thereafter, in collaboration with the medical records officers, the case notes of all new patients presenting for antenatal care at 20 - 32 weeks gestational age will be selected. Group counselling is then conducted for the volunteers. Their haemoglobin concentration (Hb conc) is then determined with a hemoglobinometer (Hemocue), using a pin-prick capillary sample and results are obtained within 5 to 10 minutes. All those with Hb conc above 10g/dl will be disqualified, and only pregnant women with Hb conc below 30% will be recruited. All the women who qualify for this study will then be given individual counselling. During this counselling session, the IVON trial will be explained to the woman in greater detail while giving her opportunities to ask questions. She will also be asked questions to confirm her understanding of all that has been said. The benefits of the clinical trial and the participant’s role in the trial will be clearly explained to her. More questions will be asked to further confirm her eligibility before considering her for recruitment if she gives an informed consent.

Once she gives a verbal consent, the informed consent form is given to her to read through. She will be given at least 15 minutes to read through the documents properly; after which all her questions and further information she might request will be addressed. When she is fully satisfied and convinced to participate in the trial, the consent forms will be administered. She signs in the presence of the research team member administering the consent. After signing, she will be given a copy of the signed consent form for her records, while another copy of the signed document will kept for IVON records.

She will then be randomised into either of the two study groups. After randomisation is completed, the randomisation code generated will be used in enrolling her on the Redcap platform. Additional information will then be obtained from her and from her case note to complete the documentation process on the IVON trial enrolment portal. Below is the consent form designed for this clinical trial.

RESPONDENTS INFORMED CONSENT FORM

**TITLE OF RESEARCH: Intravenous versus oral iron for iron deficiency anemia in pregnant Nigerian women (IVON): an open label, randomized controlled trial**

**NAME & AFFILIATION OF RESEARCHER:** The study will be coordinated by Professor Bosede B. Afolabi, the Principal Investigator in the Dept. of Obstetrics and Gynaecology, College of Medicine of University of Lagos and Lagos University Teaching Hospital, Idi-Araba, Lagos.

**INTRODUCTION:** Anaemia is a common problem in pregnancy that can affect the health of the mother or baby in a bad way and in cases of severe anaemia, can even cause death in them. Simply explained, it means that the level of red blood cells in the blood is lower than it should be. It is commoner in low income countries like ours compared to developed countries. The risk of developing anaemia during pregnancy is higher because of the changes that occur in the body system during pregnancy and the need for iron by the growing baby in the womb. In our environment, we commonly use blood tablets such as ferrous sulphate to treat anaemia if it is of mild or moderate severity and blood transfusion if severe. Many of these blood tablets have to be taken several times a day over some weeks. Some women do not tolerate these tablets well or even remember to take them. We now have some drugs to build up blood levels that can be given in drip form such as iron isomaltoside; they are safe but their use is not so popular in our environment. This study is designed to compare the effectiveness and benefits of using these drugs that can be given as drip, and the tablets we are used to, in order to decide which will be better for our women. You are invited to participate in this research because your blood level shows that you qualify to participate in this study.

**PURPOSE OF THE RESEARCH:** This study is to compare the effectiveness and benefits of using iron isomaltoside given in drip or ferrous sulphate tablet in treating iron deficiency anaemia in pregnant women.

**PROCEDURE OF THE RESEARCH:** The research involves obtaining information from you directly by asking certain questions and using your answers to complete a form designed for this study. This initial assessment will take approximately 10 minutes of your time. We will also retrieve additional information from your case note where necessary. Subsequently blood sample will be drawn from your vein at specified times from the time you join the study and at delivery and at the 6 weeks post delivery visit to check your blood and iron levels. We will update your records each time you come for your antenatal clinic. We will also follow up whenever you are on admission to monitor your progress and update your records. We will follow you up until 6 weeks after you have delivered.

**POTENTIAL BENEFITS:** The blood tests and test drugs will be given free at no cost to you all through pregnancy. You will also get your folic acid free all through pregnancy. You will have the opportunity of enjoying one-on-one contact with your health care providers as phone numbers will be exchanged. You will have the benefits of getting regular reminders of your appointments either via phone calls or text messages in order to minimize you missing antenatal visits. Generally, you will enjoy a closer monitoring of your health all through pregnancy and delivery until discharge from the hospital. The result of this research might enable us to change our current methods for treatment of iron deficiency anaemia in pregnancy. The findings will be presented at conferences in order to spread the findings to other health workers. The findings of this study will be published in reputable medical journals for wider dissemination of information.

**POTENTIAL RISKS:** This study involves collecting your information in an electronic database designed for the study and collection of blood specimen from your veins using a syringe and needle only. Therefore the harm it poses to participants is minimal. The trial drugs too have been found to be safe for use in pregnancy. You are however free to call the investigators immediately (see contact details at the end of this pamphlet) if you notice any symptom you are not sure of or fear may be a side effect of medication.

**CONFIDENTIALITY:** All information obtained in this study will be kept strictly confidential by the principal investigator. Data will be stored in passworded electronic database. Your personal details will not be released in any publication.

**WILLINGNESS TO PARTICIPATE:** Your participation in this research is entirely voluntary and if you choose not to participate, no punishment will be attached to your decision. You will not be paid any fees for participating in this research. You can choose to withdraw from the research at anytime.

**WHAT HAPPENS TO RESEARCH PARTICIPANTS AND COMMUNITY WHEN THE RESEARCH IS OVER:** The researcher will inform you of the outcome of the research if you so wish. There is no conflict of interest whatsoever.

**STATEMENT OF PERSON OBTAINING INFORMED CONSENT:**

I have fully explained this research to the respondent and given sufficient information, including the risk and benefit to make an informed decision.

Date:.................................. Signature:.............................................

**STATEMENT OF PERSON GIVING INFORMED CONSENT:**

I have read the description of the research. I understand that my participation is voluntary. I know enough about the purpose, methods, risk and benefits of the research to judge that I want to take part in it. I understand that I may freely stop being part of the study at anytime. I have received a copy of this consent form to keep for myself.

Date:.................................. Signature:.............................................

Thank you for participating in this research.

**For further enquiry, please contact:**

Researchers’ Contact:

- Prof. Bosede B. Afolabi

Mobile Contact: +2348076960670

E-mail: bbafolabi@unilag.edu.ng

Dept. of Obstetrics and Gynaecology,

College of Medicine, University of Lagos/Lagos University Teaching Hospital,

Idi-Araba, Lagos

- Prof. Hadiza Galadanci

Mobile Contact: +2348033210047

E-mail: hgaladanci@yahoo.com

Dept. of Obstetrics and Gynaecology,

Aminu Kano Teaching Hospital,

Kano

LUTH Health Research Ethics Committee’s Contact:

Room 107, Administrative Block, Lagos University Teaching Hospital,

Idi-Araba, Lagos

Aminu Kano Teaching Hospital (AKTH) Health Research Ethics Committee’s Contact:

No. 2 Zaria Road, Kano, Nigeria
